# Development of gait motor control: what happens after a sudden increase in height during adolescence?

**DOI:** 10.1186/s12938-016-0159-0

**Published:** 2016-05-20

**Authors:** Maria Cristina Bisi, Rita Stagni

**Affiliations:** Department of Electrical, Electronic and Information Engineering “Guglielmo Marconi”, University of Bologna, Viale Risorgimento 2, 40136 Bologna, Italy

**Keywords:** Adolescent growth spurt, Walking performance, Development of motor control

## Abstract

**Background:**

Basic understanding of motor control and its processes is a topic of well-known high relevance. During adolescence walking is theoretically a well-achieved fundamental skill, having reached a mature manifestation; on the other hand, adolescence is marked by a period of accelerated increases in both height and weight, referred as growth spurt. Thus, this period was chosen as a controlled and natural environment for partially isolating one of the factors influencing motor development (segment growth). The aim of the study was to compare gait performance of growing and not growing male adolescents during walking in single task (ST) and dual task (DT), in order to study which are the modifications that motor control handles when encountering a sudden change in segment length.

**Methods:**

19 adolescents were selected as growing adolescents (they showed a height increase greater than 3 cm in 3 months). A group of BMI-matched peers were selected as not growing adolescents (they showed a height increase lower than 1 cm in 3 months). Measures of acceleration of the trunk (L5 level) were collected using one tri-axial wireless inertial sensor. The participants were asked to walk at self-selected speed back and forth four times in a 10 m long corridor in ST and DT conditions. The following characteristics of gait performance were evaluated using different indices: variability, smoothness, regularity, complexity and local dynamic stability. An unpaired t-test was performed on the two groups for each method.

**Results:**

Different indices followed the hypothesized trend in the two groups, even if differences were not always statistically significant: not growing adolescents showed a lower variability and complexity of gait and a higher smoothness/rhythm. Stability results showed a similarly stable gait pattern (or even higher in DT) in the growing adolescents when compared to their not growing peers.

**Conclusions:**

The findings of the present work suggest that growth spurt affects gait variability, smoothness and regularity but not gait stability. It could be argued that sudden peripheral changes of the body affect the manifestation and the performance of gait, but, on the other hand, gait control is able to handle these modifications, maintaining the stability of the system.

## Background

Motor development is the study of the changes in human motor behaviour over the lifespan, the processes that underlie these changes, and the factors that affect them.

It is a topic of well-known high relevance among the scientific community: its comprehension is indeed fundamental in order to understand what happens in pathologic and/or elderly subjects and in order to perfect/improve individual movement performance potential by providing developmentally appropriate activities. Basic understanding of motor control and its processes is fundamental for effective interventions and therapies when problems are present [1].

Motor development has been defined as the adaptive change toward competence [1, 2] implying that adjustment, compensation and changes to reach or maintain competences continue throughout the life span. It is a discontinuous process occurring within a self-organizing control system where the development and refinement of movement patterns and skills are influenced in complex ways. Both the process and the products of motor development are indeed influenced by (i) factors within the individual (e.g. neuromotor maturation, growth rate, sensitive learning period) and (ii) factors in the environment (e.g. bonding, stimulation) operating often in conjunction.

Given the multitude of influencing factors, the correspondence between factors and effects on motor development and motor control is difficult to investigate and is still not clear.

Learning to walk is one of the most important milestones in motor development, achieved during infancy and early childhood [3]. By looking at phases and stages of motor development, walking is one of the skills achieved during the so called “fundamental movement phase” which reaches maturation around age 6–7 (mature stage). From 7 to 14 year old and up, the specialized movement phase begins (children start learning specialized movement that they can apply to specific sports and activities) which ends with the lifelong utilization stage (age 14) [1]. According to this scheme, during adolescence, walking should be a well-achieved fundamental skill, having reached a mature manifestation.

On the other hand, adolescence is marked by a period of accelerated increases in both height and weight, referred as growth spurt [1]: this period can be a controlled and natural environment for partially isolating one of the factors influencing motor development (segment growth). Given the aspects described above, adolescents theoretically have a previously organized controller that shows a mature manifestation of gait but that is suddenly affected by peripheral changes (e.g. height increase). It is indeed common to observe low gross motor coordination in this population: growth spurt can affect the output of the controller, which was previously organized on different segment dimensions. In the present work, the analysis of gait will be focused on 15 year old growing and not growing male adolescents: this choice was done in order to focus on those subjects who are experiencing a relatively late growth spurt, having had more time to reach a mature manifestation of this fundamental movement.

In order to evaluate differences between growing and not growing adolescents, parameters that evaluate variability, smoothness, regularity and stability of gait are necessary. To this purpose, literature offers several indices, often referred to as stability and variability indices [4], useful in detecting changes in gait performance, e.g. indicating adolescents that undergo a sudden growth as less stable/smooth than their not growing peers are. These types of indices are not necessarily correlated to gait stability/risk of falling, but highlight features describing different aspects of gait performance that are supposed to depend on motor control behaviour.

In particular in the present work, measures of variability (standard deviation of stride time [5], Poincaré plot [6]) measures of smoothness/rhythm (harmonic ratio [7]), measures of complexity of gait pattern (sample entropy [8, 9] and recurrence quantification analysis [10]) and direct assessment of stability [11] (short Lyapunov exponents [12]) will be included.

An advantage of using these types of indices is that, when applied on different trunk acceleration components (anteroposterior, AP, vertical, V and mediolateral, ML), they can give information about the direction/plane on which motor control is mostly affected.

In order to highlight possible differences between growing and not growing adolescents, it can be useful to perturb gait performance adding a cognitive task while walking: dual task methodology affects gait performance allowing exploring the relative cognitive demand of gait control [13, 14]. Since cognitive tasks that involve internal interfering factors seem to disturb gait performance more than those involving external interfering factors [14], a cognitive task was selected for this study.

The aim of the present study was to compare gait performance of growing and not growing male adolescents during walking in single task (ST) and dual task (DT) conditions, in order to study which are the modifications that motor control handles when encountering a relatively sudden change in segment length.

## Methods

### Study subjects

Eighty-eight 15-year old male adolescents were included in the study. All of the participants had no known developmental delay and no musculoskeletal pathology.

The Review Board Committee of the University of Bologna, “Comitato Bioetico”, approved this study (July 11th, 2012), and informed consent was obtained from the participants’ parents.

For each participant, weight and height data were collected. They were asked if they knew to have had a relevant height increase in the past few months.

Height and weight increase were monitored longitudinally every 3 months. Those who showed a height increase greater than 3 cm in 3 months were selected as the group of growing adolescents (Grown): 19 adolescents fulfilled this criterion.

Those who declared not to have had a height increase in the past months and showed a height increase lower than 1 cm in 3 months were selected as the not growing adolescents (NotGrown): 19 adolescents, fulfilling these criteria and matched by BMI with the Grown group, were selected for the NotGrown group.

Detailed information of the two groups of participants are shown in Table 1.

**Table 1:** Adolescents’ characteristics

Grown						NotGrown			
sbj	h (cm)	w (kg)	BMI	Δh (cm)	Δw (kg)	sbj	h (cm)	w (kg)	BMI
1	170.0	56.9	19.7	3.0	4.0	1	165.0	60.2	22.1
2	175.0	61.9	20.2	3.0	−1.0	2	176.0	67.1	21.6
3	175.5	50.3	16.3	3.5	1.8	3	183.0	63.6	19.0
4	171.5	51.6	17.5	3.5	3.0	4	163.0	59.0	22.2
5	173.0	92.0	30.7	3.0	0.0	5	175.5	66.0	21.4
6	163.5	51.7	19.3	3.5	3.3	6	171.5	74.6	25.4
7	166.0	47.1	17.1	3.5	2.7	7	169.5	65.7	22.9
8	173.0	62.8	21.0	3.5	−1.1	8	173.0	70.3	23.5
9	160.0	45.7	17.9	4.0	3.3	9	169.0	56.0	19.6
10	163.0	47.2	17.8	4.0	2.6	10	161.5	63.2	24.1
11	166.5	46.6	16.8	4.0	1.9	11	169.5	74.4	25.8
12	170.0	57.0	19.7	4.0	0.0	12	173.0	64.6	21.6
13	173.0	57.3	19.1	5.0	3.7	13	170.0	49.0	17.0
14	177.0	62.3	19.9	4.0	3.7	14	168.5	52.5	18.5
15	174.5	64.9	21.3	3.5	4.4	15	156.0	51.0	21.0
16	167.5	71.2	25.4	3.0	5.3	16	167.0	67.6	24.2
17	164.0	51.2	19.0	4.5	2.2	17	178.0	64.4	20.3
18	150.5	44.0	19.4	5.5	13.2	18	163.0	46.0	17.3
19	167.5	56.3	20.1	4.0	1.7	19	177.0	77.9	24.9

Adolescents’ characteristics. Height (h), weight (w) and body mass index (BMI) of the Grown and NotGrown group at session 1. Δh (cm) and Δw (kg) are the increments in height and weight found in the Grown group

### Experimental setup

Three tri-axial wireless inertial sensors (OPALS, Apdm, USA) were mounted respectively on the lower back (at L5 level) and on the lower legs using straps. Sensors characteristics: accelerometer and gyroscope noise, respectively 0.0012 m/s^2^/√Hz and 0.05 deg/s/√Hz, sensors dimensions, 48.4 × 36.1 × 13.4 mm (L × W × H), weight, <22 g (with battery).

Measures of accelerations of the trunk and of the legs were recorded at 128 Hz. The participants were asked to walk at self-selected speed back and forth in a 10-meter long corridor for a total of 40 m walk, in two conditions: free walking and walking performing a cognitive DT.

The cognitive DT consisted in counting backward aloud by eights from a random starting number.

Trials were also video recorded in order to check if the task was correctly performed (e.g. to identify distractions, laughs etc.). In those cases, the identified steps were excluded from the analysis.

All the participants performed trials during the first session (session 1). The Grown group repeated the test after 3 months (session 2), when the sudden increase in height was found.

### Data analysis

Stride detection was estimated from the angular velocity around the medio-lateral axis of the ankle [15]; stride time was defined as the time elapsed between the first contact of two consecutive footsteps of the same foot. The turns, the first two and the last two strides of each trial were excluded from the analysis. For all the participants and each condition, 20 strides were analyzed.

Raw unfiltered data were analyzed to assure that information was not lost or altered due to filtering. Matlab R2012a (MathWorks BV, USA) was used for data and statistical analysis.

The following methods for gait pattern evaluation were used [4]:

1. Variability measures:
  Standard deviation of stride time (STv) [5];
  Short term variability of stride estimated via Poincaré plots (SD1) [6];
2. Smoothness/regularity/complexity measures:
  Harmonic ratio (HR) [16, 17]: HR was estimated on the three L5 acceleration signal components (HRv, HRap, HRml);
  Sample entropy (SE) [8]: SE was calculated on the V, AP and ML accelerations of L5 (SEv, SEap and SEml) for values of τ ranging from 1 to 6 (following a multiscale entropy approach [18]).
  Recurrence quantification analysis (RQA) [10, 19, 20]. Recurrence rate (RR), determinism (DET), averaged diagonal line length (AvgL) were calculated applying the method on the V, AP and ML accelerations of L5.
3. Stability measures:
  Short term Lyapunov exponents [12, 21] (sLE): sLE were calculated using 4 different state spaces compositions, one composed by the three linear acceleration components of the trunk (sLE3) and three composed by the delay embedded state spaces of one acceleration (sLEv, sLEap and sLEml).

Each method was applied on each participant’s test in the two walking conditions (ST and DT). Detailed implementation of the listed methods is presented in [4].

A Jarque-Bera test [22] was performed to test normal distributions of the estimated parameters on the different groups: since normal distribution was verified on all the groups, mean and standard deviation of gait parameters were calculated for each group.

An unpaired Student’s t test (level of significance 0.1) was performed on the two groups for each method: t test was right- or left-tailed hypothesizing higher stability/smoothness/repeatability in the NotGrown group.

## Results

### Variability measures

During ST gait, STv and SD1 did not show statistically significant difference between the NotGrown and the Grown groups. When analysing DT gait, STv and SD1 followed the expected trend and were statistically lower in the NotGrown group when compared to the Grown group. STv and SD1 were always higher during DT gait when compared to ST gait.

### Smoothness/regularity/complexity measures

Harmonic ratio during ST gait showed similar mean values for the two groups when applied on the V and ML acceleration components. HRap was statistically higher in the NotGrown group (2.7 ± 0.5) than in the Grown group (2.4 ± 0.4), as hypothesized. In the DT condition, HR resulted statistically higher in the NotGrown group when compared to the Grown group on all the 3 axes (HRv, HRml and HRap). In general, when comparing ST and DT gait, HR values during DT were lower than during ST gait.

When analysing SE results, the t-test showed statistically significant differences only when SE was calculated on the AP axis with τ = 6 in ST condition and on the V axis with τ ≥ 2 in DT condition. No differences between groups were shown on the ML axis. SE results during DT were in general close to or slightly lower than SE values obtained on ST gait.

RQA values followed the hypothesized trend and showed statistically significant differences between the two groups when applied on the ML and AP acceleration components in ST gait (RRap, DETap, DETml, AvgLap and AvgLml) and on the AP direction during DT gait (RRap). RQA mean results, calculated on the ML and AP directions, were higher in DT gait when compared to ST gait.

### Stability measures

sLE did not show statistically significant differences between the two groups in ST gait. In DT gait, NotGrown sLEv showed mean values statistically higher than the Grown ones, not following the expected trend of higher stability in the growing adolescents. sLE during DT gait were close to or higher than the ones estimated in ST gait.

Table 2 show mean and standard deviation of obtained results for the two groups (NotGrown and Grown) in ST and DT condition. Asterisks indicate statistical differences found between the two groups.

**Table 2:** Gait performance results

**ST gait**						**DT gait**					
	**NotGrown**		**Grown**		p-value		**NotGrown**		**Grown**		p-value
	mean	std	mean	std			mean	std	mean	std	
SD1	0.02	0.01	0.02	0.01		SD1	0.03	0.01	0.04	0.02	*0.02
STv	0.03	0.01	0.03	0.01		STv	0.04	0.02	0.07	0.05	*0.03
HRv	3.11	0.52	3.11	0.53		HRv	3.01	0.56	2.73	0.53	*0.06
HRml	2.29	0.54	2.2	0.43		HRml	2.27	0.39	2.01	0.33	*0.02
HRap	2.66	0.48	2.37	0.4	*0.03	HRap	2.46	0.46	2.3	0.45	*0.1
SEap τ=1	0.44	0.07	0.45	0.07		SEap τ=1	0.43	0.06	0.44	0.07	
SEap τ=2	0.68	0.11	0.7	0.12		SEap τ=2	0.68	0.1	0.68	0.12	
SEap τ=3	0.87	0.14	0.89	0.16		SEap τ=3	0.85	0.12	0.86	0.16	
SEap τ=4	0.99	0.17	1.02	0.17		SEap τ=4	0.98	0.12	0.98	0.18	
SEap τ=5	1.09	0.18	1.14	0.19		SEap τ=5	1.06	0.14	1.08	0.18	
SEap τ=6	1.13	0.16	1.21	0.18	*0.09	SEap τ=6	1.12	0.17	1.16	0.18	
SEv τ=1	0.45	0.07	0.46	0.07		SEv τ=1	0.42	0.07	0.44	0.07	
SEv τ=2	0.67	0.11	0.71	0.1		SEv τ=2	0.63	0.11	0.67	0.1	*0.1
SEv τ=3	0.88	0.15	0.93	0.14		SEv τ=3	0.82	0.14	0.88	0.15	*0.1
SEv τ=4	1.06	0.18	1.09	0.14		SEv τ=4	0.99	0.18	1.04	0.18	*0.1
SEv τ=5	1.19	0.23	1.2	0.16		SEv τ=5	1.09	0.18	1.16	0.17	*0.1
SEv τ=6	1.28	0.2	1.27	0.16		SEv τ=6	1.18	0.19	1.25	0.17	*0.1
SEml τ=1	0.57	0.06	0.57	0.09		SEml τ=1	0.55	0.06	0.54	0.09	
SEml τ=2	0.86	0.09	0.89	0.15		SEml τ=2	0.84	0.11	0.85	0.15	
SEml τ=3	1.19	0.12	1.21	0.2		SEml τ=3	1.13	0.16	1.16	0.22	
SEml τ=4	1.46	0.14	1.45	0.22		SEml τ=4	1.37	0.18	1.39	0.24	
SEml τ=5	1.63	0.14	1.62	0.22		SEml τ=5	1.51	0.16	1.56	0.27	
SEml τ=6	1.72	0.15	1.72	0.24		SEml τ=6	1.61	0.15	1.63	0.26	
RRv	16.02	2.03	16.43	2.88		RRv	15.99	1.89	15.56	2.05	
DETv	92.18	12	92.19	11.91		DETv	94.48	9.5	94.69	11.42	
AvgLv	9.01	1.67	9.07	1.71		AvgLv	9.17	1.83	9.13	2.05	
RRml	10.9	1.28	10.78	1.16		RRml	11.33	1.92	11.47	1.94	
DETml	63.36	10.38	58.01	12.83	*0.09	DETml	69.73	11.42	66.82	13.61	
AvgLml	6.51	0.57	6.07	0.56	*0.01	AvgLml	6.93	0.9	6.91	1.35	
RRap	19.47	1.55	18.16	1.98	*0.01	RRap	20.21	1.6	19.17	2.27	*0.06
DETap	79.16	13.09	73.28	18.54	*0.1	DETap	82.86	11.35	81.74	17.24	
AvgLap	7.75	0.92	7.28	0.96	*0.07	AvgLap	8.58	1.07	8.41	1.2	
sLE3	0.4	0.11	0.37	0.09		sLE3	0.46	0.09	0.43	0.1	
sLEv	1.01	0.16	0.95	0.17		sLEv	1.07	0.19	0.98	0.12	*0.04
sLEml	0.88	0.2	0.8	0.2		sLEml	0.92	0.17	0.85	0.19	
sLEap	0.66	0.11	0.64	0.11		sLEap	0.76	0.13	0.79	0.17	

Mean (mean) and standard deviation (std) of gait performance results obtained for the NotGrown and the Grown group in ST (ST gait) and DT condition (DT gait). Asterisks (*) and p-values indicating significant differences between the two groups

## Discussion

In the present study, gait patterns of growing and not growing male adolescents were compared using measures of variability, stability, smoothness and complexity both in single and dual task conditions. The aim was to evaluate the modifications that motor control handles when encountering a segment length growth spurt. We hypothesized a lower performance during gait in growing adolescents due to the encountered sudden physical body changes. In order to highlight differences between the two groups, walking performance both in ST and DT condition was evaluated using the following indices: variability (STv and SD1), smoothness (HR), complexity (SE and RQA) and stability (sLE).

Our hypothesis was generally confirmed by the trend shown by the different indices in the two groups, even if differences were not always statistically significant for all the estimated parameters: not growing adolescents showed a lower variability and complexity of gait and a higher smoothness/rhythm. Stability results showed a similarly stable gait pattern (or even higher in DT gait) in the growing adolescents when compared to their not growing peers.

In ST gait, the variability of the two groups was similar, showing that the manifestation of the gait performance was not affected apparently by the growth spurt during single task. During DT, both SD1 and STv mean values of both groups were higher when compared to ST condition and were statistically higher in the Grown group when compared to the NotGrown. The difference in variability between ST and DT walking was expected and in agreement with literature [23]. The Grown group was more affected by the DT condition: literature suggests that cognitive tasks share complex neural networks connecting different brain regions [24, 25] which are interlinked with those of gait control, and the demand placed by cognitive tasks interferes with these networks disturbing gait. The results of the present study suggest that cognitive motor interference had a higher influence on growing adolescents than on their not growing peers.

HR, in ST condition, resulted to be statistically higher in the NotGrown group than in the Grown group when applied on the AP direction: these results are in agreement with those found in literature [7] that showed that older adults are less smooth than young adults in the direction of progression (AP). HR, being a measure of smoothness, can be thought as a measure of the motor control of walking [7]: if this is true, growth spurt affects gait control in the same direction as ageing. During DT, HR was in general lower than during ST. Looking at the two groups, HR was statistically higher in the NotGrown group on all the three axes analysed (V, AP and ML). These results are in agreement with other literature showing a decreased HR during DT walking of trunk acceleration of older adults in all the directions [26]. Literature suggests that a decreased HR may be caused by an adaptation of motor control to new challenges [26]: decreased HR have been reported both for walking under DT and for walking with additional challenges (e.g. walking on an irregular surface) [17, 26]. This could suggest that the decreased smoothness, in the AP direction during ST and in the three directions during DT, highlights the adaptation that adolescents' motor control have to implement to overcome the modifications in its peripheral system.

SE showed higher complexity of gait in the Grown when compared to the NotGrown when applied on the AP axis in ST gait (SEap) and on the AP and V axis (SEap and SEv) in the DT gait; in DT condition, SEap did not show statistically significant differences, but the trend was maintained for all the values of τ. Small SE values are associated with great regularity while large SE values represent a small chance of similar data being repeated: in this case, the regularity of gait pattern was always higher in the NotGrown adolescents on the sagittal plane. These results are in agreement with what was shown by Lamoth et al. [9] who did not find differences in the ML trunk acceleration time-series. SE results during DT condition were in general lower than in ST condition: these results are not in agreement with previous work on elderly individuals, Lamoth et al. [9] found that during DT (a letter fluency task), SE showed larger values in elderly individuals indicating that changes in cognitive functions contribute to changes in gait stability and automaticity. In adolescent population, the trend was opposite: SE results showed a small decrease in gait complexity (and an increase in regularity) during DT. This trend, even if not expected, could be explained by a more automatic way of walking that adolescents use while performing the cognitive task: their strategy is probably different to the one used by the elderly population studied in the work by Lamoth et al. [9]. To authors’ knowledge, there is no other literature evaluating SE during DT: more research is needed to further analyse the effect of DT walking on this index in order to further explain its physiological meaning.

RQA showed differences in ST condition on ML and AP axis: DET and AvgL (on AP and ML) and RRap were lower in the Grown group than in the NotGrown during ST gait. In DT gait, only RRap was higher in the Grown than in the NotGrown. Recurrence quantification analysis aims to evaluate walking performance assessing postural control processes during locomotion [10]: the statistically significant differences found in ST gait highlighted that trunk accelerations in AP and ML direction were more chaotic in the Grown group than in the NotGrown and that growing adolescents showed a lower balance performance. DT condition reduced the differences in RQA results between the two groups, showing a similar performance of the postural control during DT walking.

sLE showed no statistically significant differences between the two groups in ST gait, while local dynamic stability was statistically higher in the Grown when compared to the NotGrown when analysing the V acceleration component in DT gait. The shown trend was not in agreement with the hypothesis of this work on stability: we expected to see a more stable gait pattern in the not growing adolescents when compared to growing peers. These results suggest that growing adolescents, despite higher variability and lower regularity of their gait, achieve a high level of gait stability, possibly using a higher amount of involvement of central control during the task. It could be suggested that the Grown increase in SE highlights an increase of cognitive input during walking [27], that, in perfectly healthy population can lead to an increase in stability. In general, sLE during DT gait were close to or higher than the ones in the ST gait, in agreement with what was previously found on elderlies [4, 9].

In general, small differences between the Grown and NotGrown have been shown: this result could have been expected because both groups were composed by healthy young subjects, conducting a well-achieved fundamental skill. In future, based on the results of this study, the evaluation of the performance during different coordination exercises (e.g. looking separately at static and dynamic coordination) could further highlight which aspects of motor control are mostly affected by growth spurt.

It has also to be acknowledged that, given the many comparisons performed simultaneously, there is the possibility of type I errors (multiple comparison problem). On the other hand, in the present study, several comparisons were conducted to investigate different aspects of gait control and, when assessing similar aspects (e.g. variability), the same trends were obtained from different parameters, thus reinforcing the found results. In addition, even when the results of the two groups were not statistically different, obtained values followed the hypothesized trend, thus supporting the physiological meaning of the shown differences. Future works will have to focus on the identified trends in order to test them furtherly on different and more numerous groups of participants.

In conclusion, the findings of the present work suggest that growth spurt affects gait variability, smoothness and regularity but not gait stability. It could be argued that sudden peripheral changes of the body affect the manifestation and the performance of gait, but, on the other hand, gait control is able to handle these modifications, maintaining the stability of the system. These results confirmed that in healthy young subjects, gait variability is not always an indirect assessment of stability because central control can handle variable gait manifestations in a controlled and stable way [28].

In the present study, differences in gait performance of Grown and NotGrown groups were related to sudden changes in segment length: certainly, growth spurt was one relevant and theoretically predominant influencing factor of motor development in the analysed subjects, but it was probably acting concurrently with other different factors. The considered population is indeed experiencing a very peculiar period of transition from childhood to adulthood that involves biological, cognitive and socio-emotional changes [29], that concur in affecting motor development. Nevertheless, the results of the present work add relevant considerations and observations to basic knowledge of motor development: in order to further understand motor control, more research on adolescent movement performance is necessary, with particular focus on how physical and cognitive changes affect motor development [1].

## Conclusion

The findings of the present work suggest that growth spurt during adolescence affects variability, smoothness and regularity of gait but not its stability. Sudden peripheral changes of the body happening in growing adolescents affected the manifestation and the performance of gait in the analysed population; on the other hand, gait control of young healthy growing subjects was able to handle these modifications, maintaining a level of gait stability close to their not growing peers.
